# Editorial: Insights in obesity: 2023

**DOI:** 10.3389/fendo.2024.1489087

**Published:** 2024-11-13

**Authors:** Katsunori Nonogaki

**Affiliations:** Division of Diabetes and Nutrition, RARiS, Tohoku University, Sendai, Miyagi, Japan

**Keywords:** obesity, GLP-1, GIP, semaglutide, stigma, inheritance, COVID-19

Obesity is a health condition characterized by excessive body fat that can contribute to the deterioration of other health conditions. Despite preventive lifestyle interventions, the prevalence of obesity continues to increase worldwide. According to the World Health Organization, obesity rates have more than doubled in adults and quadrupled in children and adolescents (5–19 years of age) worldwide since 1990. The World Health Statistics 2022 report indicated that 2.5 billion adults (18 years and older) and more than 390 million children were overweight. Of these, 890 million adults and 160 million children were living with obesity.

In this Research Topic, Sivakumar et al. reviewed the current evidence and molecular mechanisms underlying the inheritance of obesity, with particular emphasis on transgenerational inheritance. Evidence from epidemiologic and murine studies links parental obesity to increased weight in the offspring and cardiometabolic complications in adulthood. The authors suggest the importance of maternal influence on offspring weight and health.

Li et al. reported the impact of the COVID-19 pandemic lockdown on body mass index (BMI) in 6156 Chinese college students in a three-year cohort follow-up study from 2019 to 2021. BMI increased both during and after lockdown periods among Chinese college students. Individuals with a higher BMI exhibited a diminished BMI growth rate during the lockdown but had an accelerated BMI growth rate after the lockdown compared to those with a lower BMI.

Obesity is typically diagnosed by calculating the BMI: weight (kg)/height² (m²). In this Research Topic, novel indices were used to evaluate the prevalence of obesity-related health conditions.

A number of manuscripts in this Research Topic examined cross-sectional relationships between self-reported medical conditions and correlates of obesity and central obesity (Liu et al. and Nan et al.). Whilst interesting, these associations do not validate the anthropometric parameters examined nor establish any causative relationship with the often self-reported conditions. Evidence requires longitudinal data with, most importantly, rigorous ascertainment of the condition endpoint and covariates. Further, accepted contemporary epidemiological statistical methodologies are necessary, such as linear mixed models. Therefore, much remains to be answered about the relationship between obesity, body fat distribution, and other conditions.

Pharmacologic agents for weight loss in obesity and health outcomes were recently developed. In 2021, the US Food and Drug Administration (FDA) approved semaglutide, a glucagon-like peptide (GLP)-1 receptor agonist (GLP-1RA) given as a once-weekly injection for weight loss in adults who are obese (BMI ≥30) or overweight (BMI ≥27) and are experiencing weight-related medical problems. The drug is used to help them lose weight and keep the weight off when used in combination with a healthy diet and routine exercise. In 2023, tirzepatide, a glucose-dependent insulinotropic peptide (GIP) and GLP-1 receptor agonist, also given as a once-weekly injection, was approved by the FDA for chronic weight management. Moreover, GLP-1 treatments in which GLP-1 activity is combined with complementary activities of glucagon or amylin are beginning to reach clinical practice. A triple-hormone-receptor agonist, Retatrutid, which activates the GIP, GLP-1, and glucagon receptors, has completed its phase 2 trial for adults with obesity.

The systematic review by Xue et al. provided an up-to-date overview of the pharmacoeconomic evaluation of five anti-obesity drugs approved by the FDA for chronic weight management, namely orlistat, phentermine/topiramate, naltrexone/bupropion, liraglutide 3.0 mg, and semaglutide 2.4 mg. Studies revealed that semaglutide 2.4 mg injection exhibited more favorable cost-effectiveness than the other treatments.

Tzoulis et al. raised key unresolved questions about semaglutide for weight loss, highlighting the need for prospective studies, both controlled and real-world, regarding the optimal duration of treatment, predictors of response, appropriate lifestyle interventions, long-term safety profile, and its use in specific settings. GLP-1RAs have beneficial actions beyond glucose management and weight loss, such as reducing the risk of heart and kidney diseases. In March 2024, the FDA approved an additional indication for semaglutide to reduce the risk of major cardiovascular events such as death, heart attack, or stroke in adults with known heart disease and who are either obese or overweight in conjunction with a reduced-calorie diet and increased physical activity. On the other hand, GLP-1RAs are also associated with adverse events, including nausea, vomiting, constipation, diarrhea, gallbladder disease, and acute kidney injury. Once-weekly GLP-1 medicines have increased interest in the long-term efficacy and safety of GLP-1 medicines.

Regarding the psychosocial conditions of obesity, Sanchez et al. assessed the obesity stigma and discrimination among Spanish populations in a cross-sectional observational study. Negative attitudes towards 1018 people with obesity were assessed through BMI and three questionnaires: the Antifat Attitude Scale, Stigmatizing Situations Inventory, and Weight Bias Internationalization. All questionnaires showed a progressive increase in scores from participants of a normal weight to those with obesity. Particularly noteworthy is that bariatric surgery did not improve the social stigma scores. Future investigations are expected to evaluate the impact of GLP-1 medicines on psychosocial conditions, including obesity stigma, depression, and suicidality risk.

